# 

**Published:** 1882-07

**Authors:** 


CONTENTS.

Pneumonia....343
Care of Children....345
Health Capacity....345
Facts Worth Remembering....346
A Lay Sermon....348
Scientific Notes....350
Sea-Weed as an Anti-Fat....350
Feeding Horses....351
Pure Water....353
Servant Girls....356
Human Health....357
Vermin Riddance....358
Intussusception....358
Ventilation....359
Disease and Benevolence....360
Insanity....361
Civilization and Health....364
Equanimity of Mind....370
Why?....372
Restless Wanderers....373
The Aim of Life....374
Our Children's Teachers....376
Thermometers....377
Style....378
Influence of Temper on Health....379
The Loss of Children....379
Kindness the Best Punishment....382
Cheapest Food....383
The Grave of Hope....383
Lead Poison....386
Prostitution of High Health....387
The Human Teeth....388
Politics and Physic....389
Bequests to Children....392
Sleep....392
Imprudence *versus* Climate....393
Nursing Animosities....394
Finger Nails....394
Checked Perspiration....395
Remedy for Cough....397
Throat Ail....400
Influences....402

